# Targeted Inhibition of mGlu5 Receptors in the Contralesional Hemisphere Improves Functional Recovery After Stroke

**DOI:** 10.1161/STROKEAHA.125.054261

**Published:** 2026-02-18

**Authors:** Federica Mastroiacovo, Serena Notartomaso, Slavianka Georgieva Moyanova, Amadeu Llebaria, Xavier Gómez-Santacana, Domenico Bucci, Valeria Bruno, Giuseppe Battaglia, Karsten Ruscher, Adam Q. Bauer, Tadeusz Wieloch, Ferdinando Nicoletti

**Affiliations:** Department of Molecular Pathology, Istituto di Ricovero e Cura a Carattere Scientifico (IRCCS) Neuromed, Pozzilli, Italy (F.M., S.N., S.G.M., D.B., V.B., G.B., F.N.).; Department of Medicinal Chemistry and Synthesis, Institute for Advanced Chemistry of Catalonia (IQAC-CSIC), Barcelona, Spain (A.L., X.G.-S.).; Department of Physiology and Pharmacology, Sapienza University, Rome, Italy (V.B., G.B., F.N.).; Division of Neurosurgery, Laboratory for Experimental Brain Research, Department of Clinical Sciences, Lund University, Sweden (K.R., T.W.).; Department of Radiology, Washington University in St. Louis, MO (A.Q.B.).

**Keywords:** adults, cerebral arteries, ischemic stroke, risk factors, somatosensory cortex

## Abstract

**BACKGROUND:**

Understanding circuit-level changes that either enhance or impair the brain’s capacity for recovery will inform the design of more specific, targeted interventions to enhance recovery from stroke. We previously reported that pharmacological blockade of mGlu5 (type-5 metabotropic glutamate) receptors improves recovery of sensorimotor function in rodent models of stroke, concomitant with restoration of functional connectivity in the sensorimotor cortex contralateral to the infarct. Here, we applied photopharmacology and light-activatable/deactivatable mGlu5 receptor negative allosteric modulators (NAMs) to localize when and where in the brain the recovery-enhancing effects occur from systemically administered mGlu5 receptor NAMs.

**METHODS:**

Stroke was induced in C57Bl/6 mice by permanent middle cerebral artery occlusion. Mice were treated with either JF-NP-26 (7-(Diethylamino)-2-oxo-2H‑chromen‑4‑yl)methyl (2‑((3‑fluorophenyl)ethynyl)‑4,6‑dimethylpyridin‑3‑yl)carbamate) or alloswitch-1. JF-NP-26 is a caged derivative of the mGlu5 receptor NAM, raseglurant, inactive on its own, and can be activated by visible light of 405 nm. Alloswitch-1 is an active mGlu5 receptor NAM that can be inactivated by light of 405 nm and subsequently reactivated by light of 520 nm.

**RESULTS:**

Permanent middle cerebral artery occlusion caused a sensorimotor deficit measured by 2 behavioral tests. Systemic administration of alloswitch-1 either 30 minutes or 48 hours after stroke enhanced recovery. This effect was rapidly abrogated when deactivating light was delivered to the contralateral somatosensory cortex and was subsequently restored by light-induced reactivation in the same region. No recovery-enhancing effects were observed when alloswitch-1 was activated or deactivated in the ipsilateral tissue. Specific light-induced activation of JF-NP-26 in the homotopic contralateral but not the ipsilateral somatosensory cortex enhanced functional recovery within 5 minutes after irradiation. None of the treatments changed infarct sizes.

**CONCLUSIONS:**

These findings demonstrate that the homotopic contralateral somatosensory cortex is a key site of action of systemic mGlu5 receptor NAMs in enhancing restorative processes important for recovery after stroke. Targeted, light-modulated drugs represent a potential future therapeutic strategy to enhance recovery of function after stroke.

Ischemic stroke affects ≈800 000 people in the United States and is a major cause of acquired long-term disability in adults.^1^ Recovery after stroke is often incomplete, with most patients reporting chronic motor^2^ and somatosensory^3^ deficits. Beyond rehabilitative therapy, the lack of postacute restorative treatments has prompted a search for recovery-enhancing interventions. To date, large clinical trials after stroke have been unsuccessful, so the need for novel postacute stroke rehabilitative therapies remains.^4,5^ Brain dysfunction after stroke is due to a combination of initial structural damage and more widespread disruption within functional networks connected to the site of injury.^6–9^ Brain dysfunction in areas remote from the lesion has been termed diaschisis^10^ or connectomal diaschisis within the context of widespread changes in functional brain organization.^8,9,11–13^ Brain networks affected by connectomal diaschisis potentially provide the substrate for behavioral recovery after stroke. Thus, it is necessary to consider processes that might inhibit or interfere with endogenous mechanisms of systems-level brain repair^14^ and reconnection.

mGlu5 (type-5 metabotropic glutamate) receptors are postsynaptic receptors coupled to G_q/11_ protein, and their activation stimulates polyphosphoinositide hydrolysis with formation of inositol-1,4,5-trisphosphate and diacylglycerol. The resulting increase in intracellular free Ca^2+^ concentrations and protein kinase C activation drives a chain of intracellular reactions leading to long-lasting changes in synaptic function, such as long-term potentiation and long-term depression of excitatory synaptic transmission.^15^ After stroke, mGlu5 receptors might be associated with maladaptive synaptic plasticity, possibly by aberrant AMPA (ɑ-amino-3-hydroxy-5-methyl-isoxazoleproprionic acid) receptor internalization,^16^ or connectomal diaschisis (network-level disconnection) linked with neurological dysfunction.^7–9^ For example, after photothrombosis of the left somatosensory forepaw cortex in mice, homotopic contralateral excitation suppresses spontaneous circuit repair and global network reconnections necessary for behavioral recovery.^17^ These observations were associated with increased expression of *Grm5 (glutamate metabotropic receptor 5*) , the gene coding for mGlu5 receptors within perilesional tissue.^17^

In mouse or rat models of brain focal ischemia, poststroke treatment with a battery of mGlu5 receptor negative allosteric modulators (NAMs) improved lost sensorimotor functions. Importantly, blocking mGlu5 receptor activity after stroke had synergistic effects when paired with multisensory stimulation, while activation of the receptor with a positive allosteric modulator prevented the recovery-enhancing effect.^18^ Recovery enhancement from mGlu5 receptor NAMs exhibited 2 distinct temporal processes. Within an hour of administering an mGlu5 receptor NAM, animals exhibited a transient reversible recovery lasting a couple of hours. Subsequent daily treatments induced slower but persistent recovery processes observed after 3 to 5 days of treatment and progressed over 2 weeks poststroke.^18^ At the network level, poststroke treatment with mGlu5 receptor NAMs partially reversed deficits in resting state functional connectivity within the affected somatomotor network but also restored more global brain network organization in a manner more like that of healthy mice.^18^ Behavioral recovery after mGlu5 receptor NAM treatment appeared to result from acute or subacute remodeling of functional brain network organization in a manner that alleviates connectomal diaschisis. However, whether mGlu5 NAMs acted in the ipsilesional or contralesional hemisphere was not determined.^18^

We hypothesized that recovery of sensorimotor function after systemic mGlu5 receptor NAMs results from their effects in the contralateral hemisphere. Here, we tested this hypothesis using photopharmacology, a technique incorporating light-sensitive compounds that can be either activated or inactivated in response to spatially targeted illumination at appropriate wavelengths.^19^

Photopharmacology provides tight temporal and spatial control of drug activity without genetic manipulation of their receptors.^20–22^ Also, the use of light in the visible spectrum is advantageous because, as opposed to ultraviolet light, it does not damage brain tissue.

We used 2 light-sensitive mGlu5 receptor NAMs: (1) a caged-derivative of raseglurant (JF-NP-26 [7-(Diethylamino)-2-oxo-2H‑chromen‑4‑yl)methyl (2‑((3‑fluorophenyl)ethynyl)‑4,6‑dimethylpyridin‑3‑yl)carbamate]), which is inactive on its own and is activated by visible light (405 nm); and (2) the photoswitchable compound, alloswitch-1, which is an active mGlu5 NAM on its own, can be inactivated by 405 nm light, and reactivated with 525 nm light.^22,23^ The use of JF-NP-26 allows for demonstrating whether regional mGlu5 receptor blockade is sufficient to cause a biological effect, whereas alloswitch-1 allows for determining whether regional mGlu5 receptor blockade is necessary for the biological effect of systemically administered drug.

## Methods

### Data Availability

All data are available and can be provided on request.

### Drug

Light-sensitive mGlu5 receptor NAMs^22,23^: (1) JF-NP-26 (10 mg/kg, IP in 6% dimethyl sulfoxide, 6% Tween 80 in saline) and (2) alloswitch-1 (10 mg/kg, IP in 20% dimethyl sulfoxide, 20% Tween 80 in saline), provided by AL (IQAC-CSIC, Barcelona, Spain).

### Animals

We used a total of 114 adult C57Bl/6 male mice (8–12 weeks old, in-house colony, Neuromed, Pozzilli, Italy), and the individual mouse was considered the experimental unit within the studies. Mice were housed on a standard 12-hour light-dark cycle (lights on at 6:00 am) under controlled conditions (temperature, 22 °C; humidity, 40%) with food and water ad libitum. Studies were performed in accordance with national (Italian Legislative Decree 26/2014) and international (European Union [EU] Directive 2010/63/EU) guidelines and regulations on animal care and use. The study was approved by the Neuromed Institutional Animal Care and Use Committee and by the Italian Ministry of Health (Autorizzazione no. 538/2022-PR), and we performed all experiments in accordance with the ARRIVE guidelines 2.0 (Animal Research: Reporting of In Vivo Experiments; Supplemental Material).^24^ Every effort was made to minimize animal suffering and reduce the number of animals used in the experiments. Animals were randomized before treatments, and investigators were blinded to treatment and group assignment. Group, effect sizes, and power were determined based on published data (see Statistical analysis and Major Resources Table in the Supplemental Material, which also reports inclusion and exclusion criteria).

### Brain Optic Fiber Implantation

The method of optopharmacology was performed, as described earlier.^19,22^ Mice were anesthetized with isoflurane (4% for induction and 2% for maintenance) in oxygen 100% and were placed in a Stoelting Kopf stereotaxic frame (Stoelting Co, Wood Dale, IL) for implantation of optic fibers (MFC_400/430-0.66_1mm_SMR_DFL; Doric Lenses Inc, Quebec, Canada) at the following stereotaxic coordinates: anterior-posterior =+0.5; mediolateral=±1.3 and dorsoventral=−1 with reference to Bregma, according to the atlas of Paxinos and Franklin.^25^ Sites of implantation corresponded to the peri-infarct region in the right hemisphere (ipsilateral side) or the corresponding contralateral region in the left hemisphere (contralateral side). Fibers were fixed using dental cement; during surgery, body temperature was monitored and maintained at 37 °C with a rectal temperature probe connected to a heating pad. Animals were allowed to recover for 4 days in single cages. We established a priori that only animals exhibiting normal behavior in the paw placement (PP) test could be included in the study. This criterion was met in 100% of the animals.

### Induction of Permanent Focal Ischemia

Permanent middle cerebral artery occlusion (pMCAO) was induced by distal electrocauterization of the middle cerebral artery (MCA).^26–28^ Mice were anesthetized with isoflurane (4% and 2% in oxygen 100% for induction and maintenance of anesthesia, respectively), and an incision was made between the outer canthus of the eye and the external auditory meatus. The temporal muscle was bisected to expose the skull, and the MCA was exposed by means of burr hole craniotomy performed using a surgical drill. A thin layer of bone was preserved to protect the dura mater and the cortical surface against mechanical damage and thermal injury, whereas the remaining bone was gently removed. The MCA was occluded by electrocoagulation, and the arrest of blood flow was visualized and confirmed by a stereomicroscope. Afterwards, muscle and skin incisions were sutured. A rectal temperature probe connected to a heating pad was used to maintain body temperature at 37 °C throughout surgery. After surgery, mice were placed in an incubator (Compact incubator, Thermo Scientific, AHSI, Bernaggio, MI, Italy) at 37 °C for 30 minutes. The animals were included in the study if they underwent successful MCA occlusion. This was achieved in all animals subjected to pMCAO, and, therefore, no animals were excluded from the experiment at this point. To minimize potential confounders in the order of treatments and behavioral evaluations, animals were identified with codes and housed in single cages.

### Treatment With the Light-Activated mGlu5 Receptor NAM, JF-NP-26

Ischemic mice were divided (n=10 per group; total number=40 mice) using the block randomization method^29^ and treated 30 minutes after pMCAO with a caged, intrinsically inactive, derivative of the mGlu5 receptor NAM, raseglurant (JF-NP-26, 10 mg/kg in 6% dimethyl sulfoxide, 6% Tween 80 in saline, IP), or with its vehicle.^19,22^ One hour after pMCAO, 2 groups of mice treated with either JF-NP-26 or vehicle were irradiated with 405±6.6 nm blue-violet light in the somatosensory cortex ipsilateral to pMCAO for 5 minutes, whereas 2 additional JF-NP-26 or vehicle-treated groups were irradiated in the homotopic contralateral cortex. The 405 nm light was administered at 2000 mA intensity and 500-Hz frequency.^19,22^ In all groups, functional deficit in the PP and grip strength tests was evaluated before pMCAO (basal), 5 minutes before (Isch preirradiation), and 5 minutes and 23 hours after irradiation (postirradiation). In another experiment, 2 groups of mice (n=9 per group; total number=18 mice) were randomized and treated intraperitoneally with either vehicle or JF-NP-26 30 minutes after pMCAO, followed, 30 minutes later, by irradiation in the homotopic contralateral cortex. In these groups, behavioral performance in the PP test was assessed up to 7 days after irradiation. No mice were excluded from this experiment. Behavioral assessment was performed by investigators blinded to the groups to which each animal was assigned.

### Treatment With the Light-Modulated mGluR5 NAM, Alloswitch-1

Ischemic mice were randomized into 2 groups and were treated 30 minutes after pMCAO with alloswitch-1 (n=12), which is intrinsically active as mGlu5 receptor NAM (10 mg/kg in 20% dimethyl sulfoxide, 20% Tween 80 in saline, IP) or vehicle (n=10; total number=22 mice). No mice were excluded from the experiment. One hour after pMCAO mice were irradiated for 5 minutes with 405 nm light in the contralateral cortex to inactivate alloswitch-1. Functional deficit was assessed 5 minutes postirradiation. The same mice were subjected to a second irradiation with green light at 520±19 to reactivate alloswitch-1. Functional deficit was assessed before pMCAO, 25 minutes after alloswitch-1/vehicle treatment, 5 minutes after the first 405 nm irradiation, and 5 minutes and 22.5 hours after the second 520 nm irradiation. Behavioral assessment was performed by investigators blinded to the groups to which each animal was assigned. All mice were killed 24 hours after ischemia, and their brains were processed for histological analysis.

In another study, vehicle or alloswitch-1 was injected intraperitoneally 48 hours after pMCAO. Inactivating and activating irradiations were delivered either in the ipsilateral or contralateral somatosensory cortex, as described above. Behavioral performance in the PP test was assessed under basal conditions (ie, before ischemia), 2 days after pMCAO, 30 minutes after alloswitch-1 administration, 5 minutes after the first inactivating irradiation, and 5 minutes after the second reactivating irradiation. Because animals show spontaneous recovery after pMCAO during the first 48 hours, selective sorting of animals was performed to ensure that both experimental groups had similar sensorimotor deficits before the interventions. Hence, only mice that showed a full deficit in the PP test at 2 days after pMCAO were recruited for these experiments (18). Starting from a total of 34 mice, we could use 28 mice. Six mice showing partial or no deficits in the PP test were excluded from the experiment. Of the 28 recruited mice, 8 mice treated with vehicle and 8 treated with alloswitch-1 were irradiated in the contralateral cortex; 6 mice treated with vehicle and 6 treated with alloswitch-1 were irradiated in the ipsilateral cortex 48 hours after pMCAO.

### PP Test

The PP test provides information on the tactile/proprioceptive response to forepaw stimulation. Animals were placed with all paws on a horizontal surface, and the head was held at a 45° angle, so that visual stimulation was prevented.^30^ The paws to be tested (ipsilateral and contralateral to the ischemic lesion) were pushed along the edge to lose contact with the surface. The ability of the animals to place the paw back onto the table surface when their paws were moved towards the edge was evaluated. We used the following score: 1—prompt placement of the paw onto the table; 0.5—incomplete or delayed placing of the paw; 0—no placing with extension of the limb and paw.^31^

### Grip Strength Test

Neuromuscular function was assessed by measuring grip strength.^32,33^ Mice voluntarily gripped a bar of the grip strength meter (2 Biological Instruments, Besozzo, VA, Italy) with either the ipsilateral fore paw (iFP) or the contralateral fore paw (cFP) and pulled it backward. The peak force of each measurement was automatically recorded in grams (g) by the device. A mean of 5 trials was used for analysis. The analyzed data were expressed as percentage of basal mean value.

### Histology

Mice were killed 24 hours after pMCAO, and the brains were fixed in Carnoy’s solution, embedded in paraffin, and sectioned at 10 µm. Sections were deparaffinized and processed for staining with thionin (Nissl staining). The analysis was performed on sections regularly spaced every 550 µm through the extension of the ischemic region. The infarct area was outlined at a magnification of ×2.5 and quantified using the Scion Image software (National Institutes of Health, Bethesda, MD). The volume was calculated by integrating the cross-sectional area of damage on each stained section and the distance between them.^34^

### Statistical Analysis

The data were collected and analyzed in a blinded manner. The sample size was calculated according to earlier studies where similar behavioral tests were used.^18^ Statistical analysis was performed using the GraphPad PRISM software, version 8.0. In all experiments, data are presented as means±SEM, and a *P*<0.05 was considered significant. A 2-tailed unpaired Student *t* test was performed for 2-group comparisons of infarct volume. For the analysis of nonparametric PP scores, we used the Mann-Whitney *U* test or the Friedman ANOVA test followed by the Dunn multiple comparison. Group differences in the grip test were evaluated by Repeated measures 2-way ANOVA followed by Tukey or Sidak multiple comparison.

## Results

### Effects of Light-Induced Activation of JF-NP-26 in the Ipsilateral or in the Homotopic Contralateral Cortex of Mice Subjected to pMCAO

**Figure 1.**
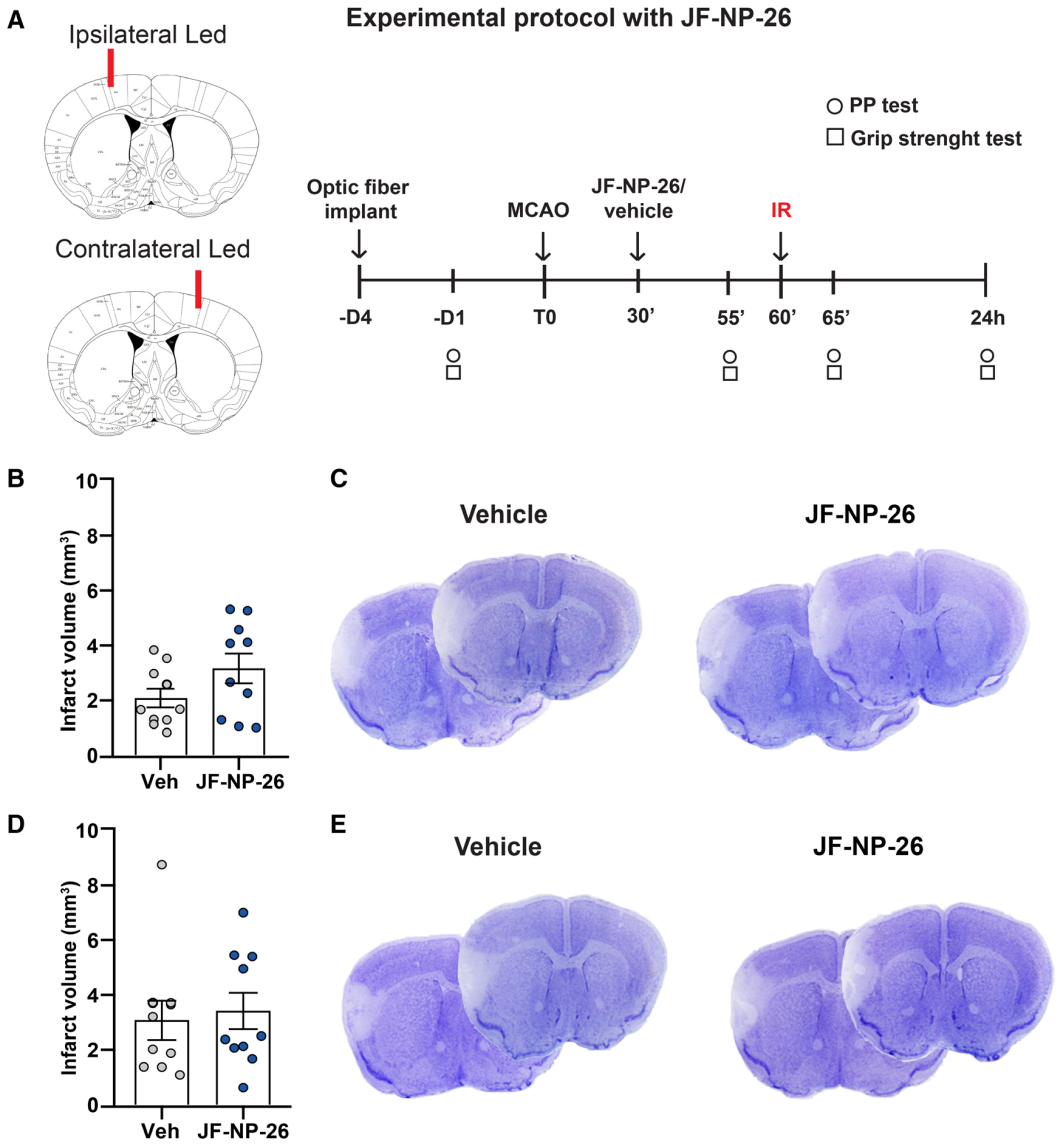
**Light-induced activation of JF-NP-26 (7-(Diethylamino)-2-oxo-2H‑chromen‑4‑yl)methyl (2‑((3‑fluorophenyl)ethynyl)‑4,6‑dimethylpyridin‑3‑yl)carbamate) in the perilesional cerebral cortex or in the homotopic contralateral hemisphere did not affect infarct volume in mice subjected to permanent middle cerebral artery occlusion (MCAO).** The experimental design is shown in **A**. Infarct volume after ipsilateral and contralateral irradiation (IR) is shown in **B**–**E**, respectively. In **B** and **D**, values are means±SEM of 10 mice per group. Led indicates light-emitting diode; PP, paw placement; and Veh, vehicle.

The experimental design is shown in Figure 1A. Mice were implanted with optic fiber (light emitting diode [LED]) in the ipsilateral cortex (peri-infarct region) or in the homotopic contralateral region 4 days before electrocoagulation of the MCA (Figure 1). The first behavioral analysis was performed 1 day before pMCAO. Mice received a single intraperitoneal injection of either JF-NP-26 (10 mg/kg) or its vehicle 30 minutes after pMCAO. Light was locally delivered 60 minutes after pMCAO (ie, 30 minutes after JN-NP-26 injections). Further behavioral analyses were performed 5 minutes before, and 5 minutes and 23 hours postirradiation. Treatment with JF-NP-26 had no effect on infarct volume compared with the vehicle group, when light was delivered either ipsilaterally (Figure 1B and 1C) or contralaterally (Figure 1D and 1E) to pMCAO. LED implantation did not affect basal PP ability and grip strength assessed 3 days after implantation (see basal columns in Supplemental Material, Figures 2 and 3; Figures S1 and S2).

**Figure 2.**
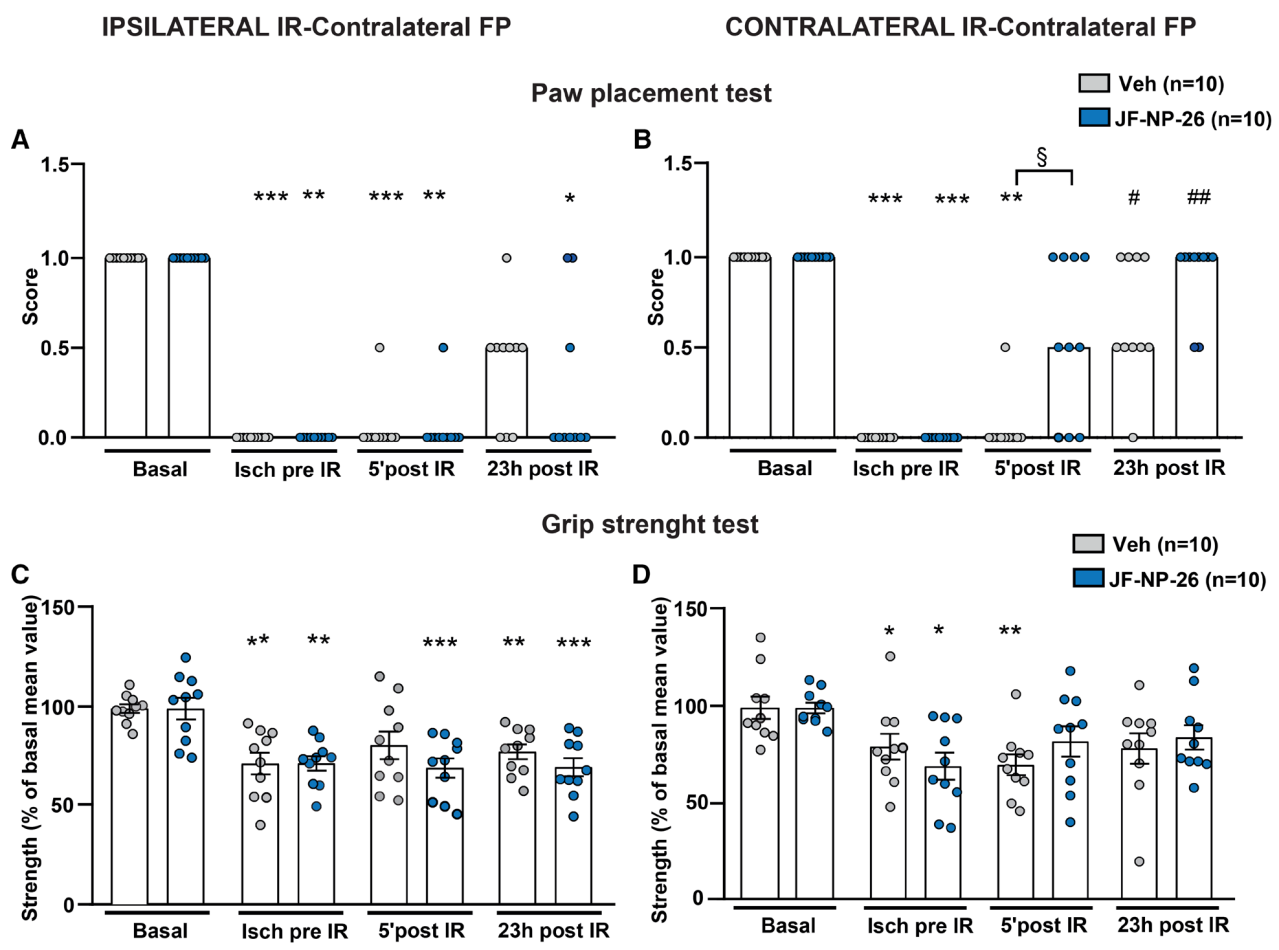
**Light-induced activation of JF-NP-26 (7-(Diethylamino)-2-oxo-2H‑chromen‑4‑yl)methyl (2‑((3‑fluorophenyl)ethynyl)‑4,6‑dimethylpyridin‑3‑yl)carbamate) in the contralateral homotopic cortex improved recovery of paw placement after permanent middle cerebral artery occlusion.** Paw placement score in the contralateral forepaw (FP) after ipsilateral or contralateral irradiation (IR) is shown in **A** and **B**, respectively. Bar graphs represent median values of 10 mice per group. Statistical analysis was performed by the Friedman test followed by Dunn multiple comparison in **A** and **B**, and Mann-Whitney nonparametric test in **B**. In **A**, time effect: Friedman statistic values=26.78 (***P<0.001 vs basal values) in vehicle (Veh)–treated mice; and 23.79 (**P<0.01 vs basal values) in JF-NP-26–treated mice. In **B**, time effect: Friedman statistic values=26.86 (***P<0.001 and **P<0.01 vs basal values; #*P*<0.05 vs pre-IR values) in Veh-treated mice; and 24.43 (***P<0.001 vs basal values; ##*P*<0.01 vs pre-IR values) in JF-NP-26–treated mice; treatment effect: Mann-Whitney *U*=18, §*P*<0.05, Veh vs JF-NP-26 at 5 minutes post IR. Grip strength test values are reported in **C** and **D** and expressed as per cent of basal value (means±SEM, n=10 mice per group). RM 2-way ANOVA+Tukey multiple comparisons: *P<0.05; ** *P*<0.01, ***P<0.001 vs the corresponding basal value; (**C**) treatment, *F*_(1,18)_=0.94, *P*=0.346; time, *F*_(2.46,44.3)_=23.12, *P*<0.0001; interaction, *F*_(3,54)_=1.14, *P*=0.34; (**D**) treatment, *F*_(1,18)_=0.1016, *P*=0.75; time, *F*_(2.32,41.76)_=9.12, *P*<0.0003; interaction, *F*_(3,54)_=1.527, *P*=0.22. Isch indicates ischemic animals.

**Figure 3.**
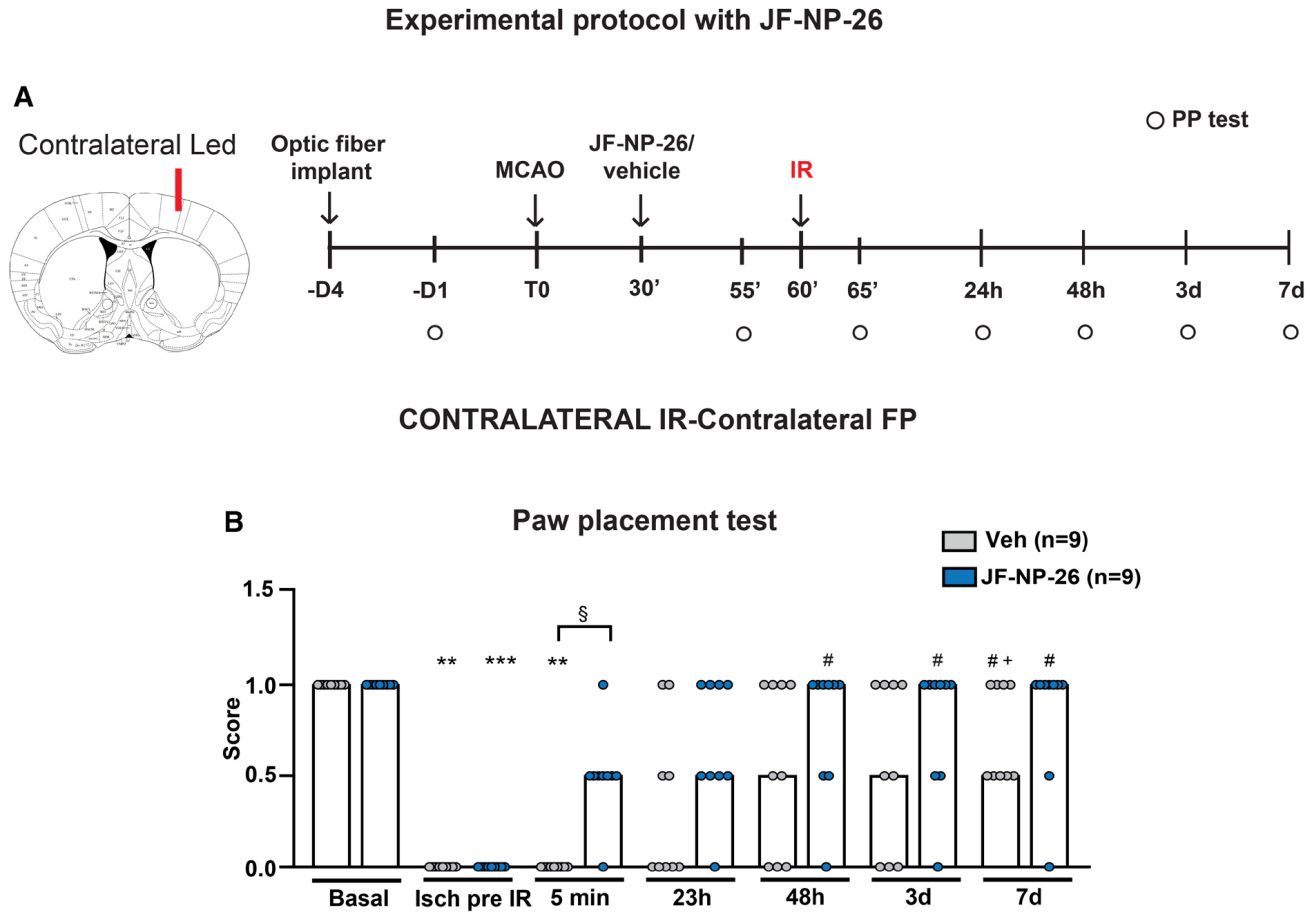
**Long-term effect of light-induced activation of JF-NP-26 (7-(Diethylamino)-2-oxo-2H‑chromen‑4‑yl)methyl (2‑((3‑fluorophenyl)ethynyl)‑4,6‑dimethylpyridin‑3‑yl)carbamate) in the contralateral homotopic cortex on functional recovery after permanent middle cerebral artery occlusion (MCAO).** The experimental design is shown in **A**. Paw placement (PP) score in the contralateral forepaw (FP) after contralateral irradiation (IR) is shown in **B**. Bar graphs represent median values of 9 mice per group. Statistical analysis was performed using the Friedman test, followed by Dunn multiple comparison and the Mann-Whitney nonparametric test in **B**. Time effect: Friedman statistic values=38.47 (**P<0.01 vs basal values; #*P*<0.05 vs pre-IR value;+*P*<0.05 vs 5 minutes) in vehicle (Veh)–treated mice; and 33.39 (***P<0.001 vs basal values; #*P*<0.05 vs pre-IR value) in JF-NP-26–treated mice; treatment effect: Mann-Whitney *U*=4.5, §*P*<0.05, Veh vs JF-NP-26 at 5 minutes post IR. Isch indicates ischemic animals; and Led, light emitting diode.

#### Effects on the Ipsilateral Forepaw Function

Behavioral analysis with the PP test showed no significant difference in the placement ability of the iFP between vehicle and JF-NP-26 injection regardless of the hemispheric position of light delivery (Figure S1A and S1B). Also, there was no difference in grip strength between the vehicle and JF-NP-26 groups at any time after start of treatment or irradiations (Figure S1C and S1D). The grip strength of the iFP decreased significantly by 14% to 21%, over 23 hours, regardless of treatment after contralateral irradiation and in the JF-NP-26–treated animals after ipsilateral irradiation, possibly due to stroke-induced microglia activation^14,35^ formation or edema formation.^36,37^ (Figure S1C and S1D).

#### Effects on the cFP Function

All mice subjected to stroke showed a severe placement deficit (PP score=0) of the cFP at 55 minutes after induction of pMCAO/25 minutes after injection of JF-NP-26 or vehicle (Figure 2A and 2B; *P*<0.001), demonstrating that inactive JF-NP-26 or vehicle per se does not affect PP. When irradiated on the lesioned ipsilateral side, the PP deficit of the cFP remained significantly depressed compared with basal levels at 5 minutes postirradiation in both treatment groups (*P*<0.01), with only 1 animal displaying moderate recovery in each group (Figure 2A). At 23 hours postirradiation, there was no difference between treatment groups. PP partially recovered in the vehicle group but was significantly lower in the JF-NP-26 group compared with the basal score (Figure 2A; *P*<0.05). In contrast, light-induced activation of JF-NP-26 in the contralateral homotopic region but not vehicle treatment facilitated the recovery of the cFP at 5 minutes postirradiation, a difference that was statistically significant (Figure 2B; *P*<0.05). At 23 hours postirradiation, there was no difference between treatment groups, though mice in the group treated with JF-NP-26 showed almost full recovery, and vehicle-treated animals showed a significant spontaneous recovery of PP compared with the preirradiation levels (Figure 2B).

Before treatments, pMCAO caused a reduction in the grip strength of cFP of 20% to 30% (*P*<0.05) in both treatment groups and irrespective of irradiation site (Figure 2C and 2D). When the ipsilateral hemisphere was irradiated, cFP grip strength transiently improved at 5 minutes postirradiation in the vehicle group but remained significantly depressed in both treatment groups at 23 hours postirradiation (Figure 2C; *P*<0.01). When the contralateral hemisphere was irradiated, JF-NP-26–treated animals improved faster than vehicle-treated animals compared with basal levels at 5 minutes postirradiation, although there was no significant difference between the treatment groups (Figure 2D).

In another experiment, we extended the time window of behavioral assessment up to 7 days in ischemic mice treated with either vehicle or JF-NP-26 30 minutes after pMCAO and irradiated with activating light exclusively in the contralateral somatosensory cortex (Figure 3). We found a significant recovery again in the PP test in mice treated with JF-NP-26 compared with vehicle-treated mice 5 minutes after delivery of activating light. At longer time intervals, vehicle-treated animals displayed a slow but persistent spontaneous recovery from 23 hours onwards after pMCAO. At these time points, the difference between JF-NP-26 and vehicle was not statistically significant, but the number of mice showing full recovery was always greater in the JF-NP-26 group at all time points, up to 7 days postirradiation. In addition, at 48 hours and 3 days after irradiation, only mice treated with JF-NP-26 showed a significant behavioral improvement with respect to preirradiation values (Figure 3). No changes in the placement ability of the iFP were found regardless of the treatment (Figure S2).

In summary, light activation of JN-NP-26, that is, local inhibition of mGlu5 receptors in the contralateral sensorimotor cortex but not in the ipsilateral perilesional cortex, improved PP function within 5 minutes of light activation.

### Effect of Light-Induced Inactivation and Reactivation of Alloswitch-1 in the Contralateral Cortex of Mice Subjected to pMCAO

**Figure 4.**
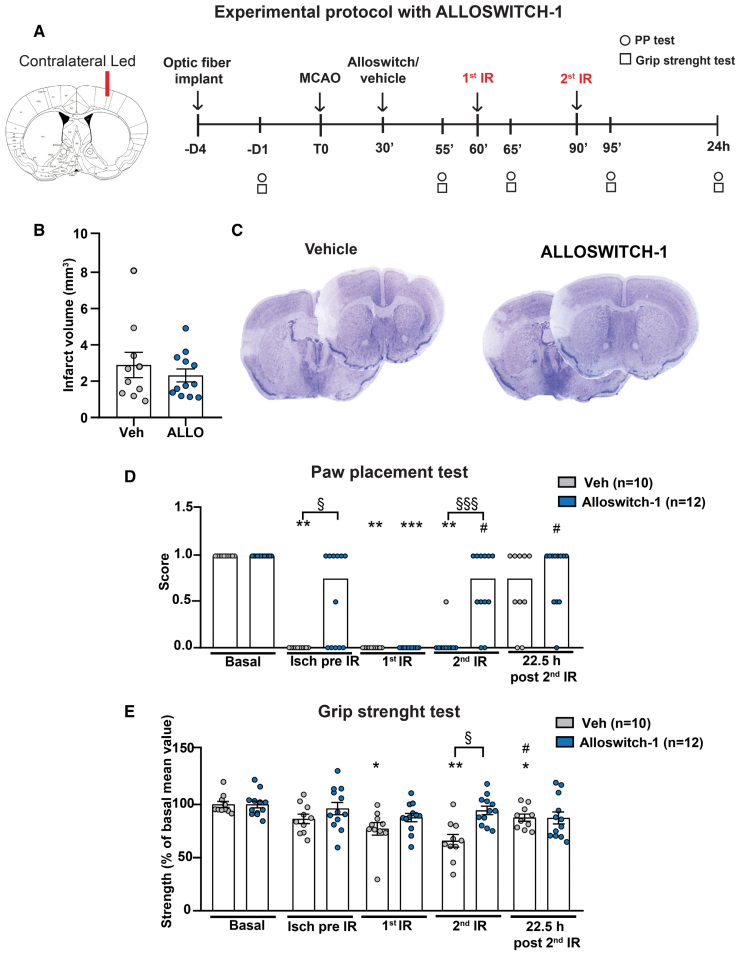
**Effect of light-induced inactivation and activation of alloswitch-1 in the contralateral homotopic cortex on functional recovery in permanent middle cerebral artery occlusion (MCAO) mice.** The experimental design is shown in **A**. Infarct volume 24 hours after contralateral inactivating and activating irradiation after vehicle (Veh) or alloswitch-1 (ALLO) treatment is shown in **B** and **C**. In **B**, values are means±SEM of 10 to 12 mice per group. Paw placement (PP) score in the contralateral forepaw (FP) after contralateral light irradiation (IR) is shown in **D**. Bar graphs represent median values of 10 to 12 mice per group. Statistical analysis was performed using the Friedman test, followed by Dunn multiple comparison and Mann-Whitney nonparametric tests. Time effect: Friedman statistic values=35.12 (**P<0.01 vs basal values) in Veh-treated mice; and 28.49 (***P<0.001 vs basal values; #*P*<0.05 vs first IR) in ALLO-treated mice; treatment effect: Mann-Whitney *U*=25 (§§*P*<0.01, Veh vs ALLO pre-IR); Mann-Whitney *U*=14.50 (§§§*P*<0.0001, Veh vs ALLO in second IR). Grip strength test in the contralateral fore paw (cFP) after contralateral IR is reported in **E** and expressed as per cent of the respective basal values (means±SEM, n=10–12 mice per group). RM 2-way ANOVA+Tukey multiple comparisons: *P<0.05; **P<0.01 vs the corresponding basal value; #*P*<0.05 vs second IR; §*P*<0.05 Veh vs ALLO after second IR (**E**) treatment, *F*_(1,20)_=4.563, *P*=0.0452; time, *F*_(2.901,58)_=8.551, *P*<0.0001; interaction, *F*_(4,80)_=4.781, *P*=0.0016. Isch indicates ischemic animals; and Led, light-emitting diode.

To examine whether mGlu5 receptor blockade in either left or right sensorimotor cortex was necessary for the beneficial effect of systemic mGlu5 receptor NAMs on functional recovery after stroke, we used the compound alloswitch-1. Alloswitch-1 is an mGlu5 receptor NAM that can be inactivated by light at 405 nm and reactivated by light at 525 nm. The experimental design is shown in Figure 4A. Mice were implanted with optic fibers in the homotopic contralateral region 4 days before the induction of ischemia, and received a single intraperitoneal injection of alloswitch-1 (10 mg/kg) or its vehicle 30 minutes after pMCAO. Inactivating light (405 nm) was delivered 60 minutes after pMCAO (ie, 30 minutes after injection of alloswitch-1 or vehicle). Reactivating light (525 nm) was delivered 30 minutes after the first irradiation (ie, 1.5 hours after pMCAO). Behavioral analysis was performed 1 hour before pMCAO, 5 minutes before and 5 minutes after the first irradiation (first irradiation), 5 minutes after the second irradiation (second irradiation), and 24 hours after pMCAO (22.5 hours postsecond irradiation). No differences in infarct volume were found between mice injected with alloswitch-1 or vehicle (Figure 4B and 4C).

#### Effects on the iFP Function

There was no difference in the PP score of the iFP between vehicle-treated and the alloswitch-1–treated groups in response to ischemia or any of the treatments (Figure S3A). Also, there were no significant differences in the grip strength of the iFP between treatment groups at any time points. As seen in Figure S1D, a decrease in grip strength of variable degree was noted in both experimental groups after irradiation compared with basal levels (Figure S3B).

#### Effects on the cFP function

The PP of the cFP 25 minutes after systemic injection of alloswitch-1 was lost in the vehicle-treated but not alloswitch-1–treated animals (Figure 4D; *P*<0.01). At this time point, the alloswitch-1–treated animals were not significantly different from basal values, signifying recovery enhancement by the mGlu5 receptor NAM. Within 5 minutes of delivery of inactivating light (405 nm; first irradiation) in the contralateral homotopic region, the recovery effect of systemic alloswitch-1 was completely abolished and was similar to vehicle-treated animals (PP=0). Five minutes after delivery of the second irradiation, the reactivating light (525 nm), PP ability was restored compared with the deficit after the first irradiation and significantly better than the vehicle group (*P*<0.001; Figure 4D). At 22.5 hours postirradiation, both alloswitch-1 and vehicle-treated animals recovered further, but only the alloswitch-1–treated animals significantly from the levels after the first irradiation. The difference between alloswitch-1 and vehicle animals was not statistically significant (Figure 4D).

The grip strength progressively and significantly decreased in the vehicle-treated group by 34% of basal values by 90 minutes post-pMCAO, which was not observed in the alloswitch-1–treated group (Figure 4E). The difference in grip strength between the vehicle (66%) and alloswitch-1 groups (94.5%) after the second reactivating irradiation with 525 nm light was significant (*P*<0.05; Figure 4E).

**Figure 5.**
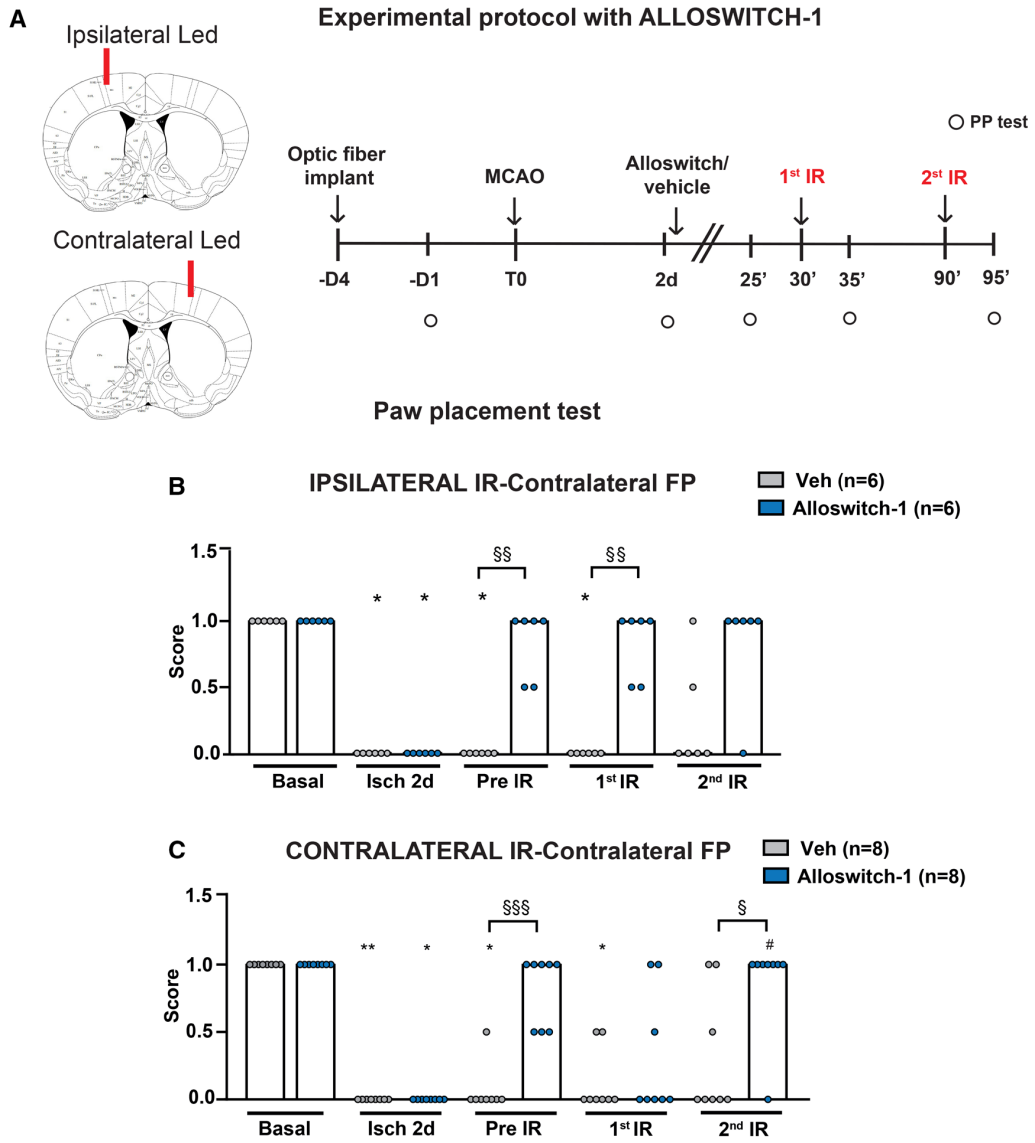
**Effect of light-induced inactivation and reactivation of alloswitch-1 systemically administered 2 days after permanent middle cerebral artery occlusion (MCAO).** The experimental design is shown in **A**. Paw placement (PP) score in the contralateral forepaw (FP) after ipsilateral (**B**) or contralateral (**C**) irradiation (IR). Statistical analysis was performed using the Friedman test, followed by Dunn multiple comparison and the Mann-Whitney nonparametric tests. In **B**, bar graphs represent median values of 6 mice per group. Time effect: Friedman statistic values=20.34 (*P<0.05 vs basal values) in vehicle (Veh)–treated mice; and 18.37 (*P<0.05 vs basal values;) in alloswitch-1–treated mice; treatment effect: Mann-Whitney *U*=0 (§§*P*<0.01, Veh vs alloswitch-1 pre-IR); Mann-Whitney *U*=0 (§§*P*<0.05, Veh vs alloswitch-1 in 1st IR). In **C**, bar graphs represent median values of 8 mice per group. Time effect: Friedman statistic values=24.19 (*P<0.05; ** *P*<0.01 vs basal values) in Veh-treated mice; and 23.83 (**P<0.01 vs basal values; #*P*<0.05 vs 2 days) in alloswitch-1–treated mice; treatment effect: Mann-Whitney *U*=1.500 (§§§*P*<0.01, Veh vs alloswitch-1 pre-IR); Mann-Whitney *U*=12.50 (§*P*<0.05, Veh vs alloswitch-1 in second IR). Isch indicates ischemic animals; and Led, light-emitting diode.

We have previously demonstrated that systemic treatment with mGluR5 NAMs starting 2 days poststroke improved functional recovery within hours after treatment.^18^ To assess in which hemisphere involves the action of mGluR5 NAM, a study with a similar experimental paradigm^18^ was performed (Figure 5A). To have comparable behavioral deficits before treatment with alloswitch-1 or vehicle, only animals with a severe PP deficit (PP score=0) at 2 days after pMCAO were included.^18^ At this time, mice were treated systemically with either vehicle or alloswitch-1, followed by inactivating and reactivating light delivered either in the contralateral or ipsilateral cortex. Hence, 25 minutes after systemic treatment with alloswitch-1, PP significantly improved compared with mice treated with vehicle-treated mice (preirradiation, Figure 5B and 5C). Neither the first inactivating light nor the subsequent reactivating light delivered in the ipsilateral somatosensory cortex affected the recovery-enhancing effect of systemic alloswitch-1 (Figure 5B). In contrast, the recovery-enhancing effect of alloswitch-1 was abolished after delivery of the first inactivating light in the contralateral cortex, and subsequently restored after delivery of the second reactivating light (Figure 5C). No changes in the placement ability of the iFP were found regardless of the treatment (Figure S4A and S4B).

In summary, treatment with alloswitch-1 restored the loss of PP function after stroke. The restored PP function was abolished within minutes of light deactivation of allowswitch-1 in the sensorimotor cortex of the contralateral but not the ipsilateral hemisphere. Subsequently, within minutes of light reactivation of alloswitch-1 in the contralateral hemisphere, sensorimotor functions recovered.

## Discussion

Using photopharmacological technology for the first time in stroke research, we identified the contralateral sensorimotor cortex as the site of action for the early recovery-enhancing effects of systemically administered mGlu5 receptor NAMs.^18^

The use of light-sensitive JF-NP-26 and alloswitch-1 allowed us to address 2 different questions regarding the recovery-enhancing effects of systemically administered mGlu5 receptor NAMs: (1) which hemisphere is responsible for the recovery-enhancing effects, and (2) is local receptor blockade sufficient to enhance recovery? Light-induced transformation of JF-NP-26 into the active NAM, raseglurant, in the contralateral homotopic cortex enhanced recovery 5 minutes after irradiation. This effect was not observed in vehicle-treated animals or after irradiating the ipsilateral cortex. Thus, pharmacological blockade of mGlu5 receptors in the contralateral homotopic somatosensory cortex is sufficient to enhance recovery after stroke.

The use of alloswitch-1 allowed us to conclude that mGlu5 receptor blockade in the contralateral homotopic cortex is necessary for the effect of systemic mGlu5 receptor NAMs on poststroke functional recovery. Systemic treatment with alloswitch-1 enhanced recovery in the PP test 30 minutes, as well as 2 days after pMCAO. The latter finding is in full agreement with the recovery-enhancing effects previously shown using different mGlu5 receptor NAMs in other rodent models of focal ischemia.^18^ The effect of alloswitch-1 was abolished by light-induced drug inactivation in the contralateral cortex and restored within 5 minutes after light-induced reactivation. In contrast, neither activating nor inhibiting light delivery in the ipsilateral cortex affected the recovery enhancement provided by alloswitch-1. Although systemic mGlu5 receptor NAMs are highly hydrophobic and cause global inhibition of mGlu5 receptors in the CNS, our results demonstrate that these compounds must block mGlu5 receptors in the contralateral cortex after stroke to enhance recovery of sensorimotor functions.

The slower recovery of PP at 23 hours postirradiation after ipsilateral activation of JF-NP-26 at 30 minutes after stroke implies that mGlu5 receptor inhibition in the peri-infarct area in isolation might hamper recovery. During this hyperacute phase after stroke, cell death processes,^14^ edema formation,^35^ and glial cell activation occur within perilesional tissue.^36,37^ In addition to neurons, mGlu5 receptors are also expressed in reactive astrocytes^38^ and microglia.^39^ Depending on the state of activation of these cells, local inhibition of mGlu5 receptors in perilesional tissue^40–42^ could differentially influence functional recovery. For example, local injection of mGlu5 receptor agonist did not affect infarct size or recovery of function up to 7 days after stroke.^42^

However, in our experiments in which alloswitch-1 was systemically injected 2 days after pMCAO, a time when acute cell death and infarct expansion have subsided, inactivating/activating irradiation in the ipsilateral sensorimotor cortex did not affect the recovery enhancement by alloswitch-1. This suggests that any recovery-hampering effect of mGlu5 receptor inhibition in the ipsilateral hemisphere is not revealed unless mGlu5 receptor blockade is local and initiated early after stroke. From a therapeutic perspective, initiation of mGlu5 receptor NAM treatment in combination with rehabilitative training is preferable days after stroke; very early mobilization of stroke patients (<24 hours after stroke) appears to be detrimental rather than beneficial.^43^

Considering the fast recovery-enhancing action of systemic mGlu5 receptor NAM treatment,^18^ we conclude that the early therapeutic effect of mGlu5 receptor NAMs on functional recovery^18^ (present data with allowswitch-1) is mediated by mGlu5 receptor blockade in the contralateral sensorimotor cortex. The contribution of the 2 hemispheres to the persistent long-term recovery enhancement by mGlu5 receptor NAM treatment^18^ remains to be assessed.

Our evidence that poststroke administration of mGlu5 receptor NAMs restored sensorimotor functions in multiple animal models of focal ischemia (in the present study and in Hakon et al^18^) raises the attractive possibility that these drugs could be used to facilitate rehabilitation in patients. mGlu5 receptor NAMs corrected the alterations in functional connectivity involving the contralateral somatosensory cortex to become more similar to healthy mice,^18^ suggesting that endogenous activation of mGlu5 receptors plays a key role in synaptic modifications that ultimately contribute to connectomal diaschisis after stroke.^7,8^ The mGlu5 receptor NAMs could exert their therapeutic activity by acting on the homotopic contralateral cortex.

Several limitations of this study present several opportunities for future experiments. We only evaluated early treatment responses to limit the experimental complexity of this study. Follow-up experiments should include prolonged mGlu5 receptor inhibition (eg, for at least 5 days) to establish phenotypes of persistent recovery after stroke. As has been demonstrated with all poststroke interventions, blocking a mechanism involved in normal signal transduction for a prolonged period might be detrimental to normal brain function. Importantly, we did not find any adverse effects of systemic administration of mGlu5 receptor NAMs in this study or when administering these compounds starting days after stroke and lasting for 2 weeks.^18^ Therefore, we have proposed that treatment with mGlu5 receptor NAMs should be initiated 2 to 5 days after stroke and continued for 2 to 4 weeks.^18^ This advice is based on similar reasoning as provided for anti-inflammatory treatments, where inflammation may be detrimental early poststroke and beneficial later during recovery. Stroke is a disease of an aging population and involves both sexes. Prior work demonstrated^18^ the restorative effects of mGlu5 receptor NAMs in female mice after stroke are of similar magnitude as those observed in males. Future work can evaluate the efficacy of mGlu5 receptor NAMs in aged animals to determine whether the effects of these compounds persist across the lifespan. Inhibiting mGlu5 receptors in the contralesional sensorimotor area was sufficient to restore PP function; however, other brain regions implicated in functional recovery (eg, visual cortex^30^ or subcortical brain regions) were not investigated.

The potential of photopharmacological approaches for site-targeted therapeutic strategies offers an exciting strategy for evaluating other circuits mediating poststroke recovery. The use of visible light is particularly advantageous compared with ultraviolet because of reduced tissue damage. As this technology improves, redshifted variants of these compounds will allow for targeting larger and possibly deeper brain regions subserving functional recovery after stroke.

## ARTICLE INFORMATION

### Sources of Funding

This work was supported by the Italian Ministry of Health (RC no. 2791719), the Alborada Trust (Dr Wieloch), Swedish Research Council (Dr Ruscher), Hans-Gabriel and Alice Trolle (HGAT)-Wachtmeisters Foundation (Drs Ruscher and Wieloch), National Institutes of Health grants R01NS126326, R01NS102870, and RF1AG07950301 (Dr Bauer).

### Disclosures

Drs Wieloch and Ruscher have ownership in Sinntaxis AB, a company developing therapies for stroke recovery, and are inventors on a patent related to the submitted study. The other authors report no conflicts.

### Supplemental Material

Supplemental Methods

Figures S1–S4

Major Resources Table

ARRIVE Checklist

## Supplementary Material


